# An explainable streaming early identification model for early neurological deterioration based on coordinated fusion of ECG waveforms and vital signs

**DOI:** 10.3389/fneur.2026.1799631

**Published:** 2026-05-07

**Authors:** Yuyan Zhang, Shihan Yao, Bo Wen, Jinjie Liu

**Affiliations:** 1 School of Computer and Communication Engineering, University of Science and Technology Beijing, Beijing, China; 2 Department of Neurology, Central Hospital of Dalian University of Technology, Dalian, China

**Keywords:** cost-sensitive learning, early neurological deterioration (END), intelligent monitoring, ischemic stroke, multimodal fusion, transformer

## Abstract

Endovascular treatment (EVT) has emerged as a cornerstone in the clinical management of stroke. However, postoperative Early Neurological Deterioration (END) persists as a formidable challenge, frequently correlating with long-term disability and poor prognosis. Consequently, timely and accurate prediction of END is imperative for guiding early clinical intervention and improving patient outcomes. This study introduces DSF-Net, a cost-sensitive, dual-stream multimodal framework engineered for the continuous postoperative monitoring of stroke patients. The architecture leverages a dual-stream design: the first stream employs a 1D convolutional neural network (1D-CNN) to extract latent representations from high-frequency physiological waveforms, while the second stream utilizes a multilayer perceptron (MLP) to encode structured clinical indicators. These fused representations are subsequently processed by a Transformer encoder to capture complex temporal dependencies within granular monitoring windows. To mitigate the challenges posed by extreme class imbalance (i.e., the scarcity of END-positive instances), we integrate cost-sensitive learning with a dynamic threshold optimization strategy prioritized for F1-score maximization. Experimental results demonstrate that DSF-Net achieves superior predictive performance on postoperative monitoring datasets, yielding an Area Under the Curve (AUC) of 0.9996 and an F1-score of 0.9841. Notably, with the optimized threshold, the model enhances the END recall rate to 99%, a significant improvement over the 74% achieved by a conventional LSTM baseline. Furthermore, interpretability analysis using Integrated Gradients (IG) reveals that the model can identify subtle morphological variations in waveforms preceding deterioration, thereby facilitating transparent and actionable early warnings. These findings suggest that DSF-Net provides a robust and interpretable solution for intelligent monitoring in post-stroke care.

## 1 Introduction

Stroke remains a leading cause of mortality and long-term disability worldwide. Despite continuous advancements in medical technology, stroke is still the second leading cause of death—subsequent only to ischemic heart disease—and the third leading cause of disability-adjusted life years lost globally (1). Recent epidemiological studies further indicate that millions of new stroke cases occur annually, accompanied by an alarming shift toward an earlier age of onset (2). In the Neurocritical Care Unit (NCU), post-stroke patients are particularly susceptible to Early Neurological Deterioration (END), an abrupt decline in neurological function that is tightly correlated with adverse clinical outcomes (3). Consequently, the timely and accurate prediction of END is of paramount clinical significance, as it provides clinicians with a narrow but critical therapeutic window for early intervention. However, current bedside practices rely heavily on intermittent neurological examinations and the periodic charting of vital signs, which often fail to capture the rapid and subtle physiological variations preceding END events.

With the widespread deployment of continuous monitoring systems in modern NCUs, massive volumes of high-frequency physiological waveforms (e.g., ECG) and structured vital-sign measurements are now routinely acquired. These multimodal data streams inherently contain a wealth of dynamic precursors to END. However, interpreting them continuously in real time remains a formidable task. Consequently, there is a pressing clinical demand for automated, data-driven early warning systems. Despite the growing success of deep learning in healthcare, applying such models to END prediction faces three primary challenges. First, clinically relevant precursors frequently manifest as brief, transient physiological abnormalities within short temporal windows, which conventional sequential models struggle to capture effectively. Second, real-world clinical datasets exhibit extreme class imbalance given the rarity of deterioration events relative to stable physiological periods (4). Third, a fixed decision threshold may become suboptimal across changing clinical environments, potentially increasing false alarms and contributing to alarm fatigue (5).

To overcome these challenges, we propose DSF-Net, a cost-sensitive dual-stream fusion network designed for the early prediction of END. The proposed framework integrates features extracted from high-frequency physiological waveforms using 1D convolutional neural networks (1D-CNNs) with structured vital-sign features processed by multilayer perceptrons (MLPs). To model short-term temporal dependencies and isolate transient abnormalities, we employ a Transformer encoder with a Self-Attention mechanism over short sliding windows. To address extreme class imbalance and improve practical usability, we further incorporate a cost-sensitive asymmetric loss together with an F1-score-driven dynamic decision threshold (τ^*^). In addition, Integrated Gradients (IG) are used to project model predictions back onto the original signal space, thereby augmenting the clinical interpretability of the model.

The main contributions of this study are summarized as follows:

1. Dual-stream multimodal modeling: we propose DSF-Net, a comprehensive prediction framework that integrates continuous physiological waveforms and structured vital signs for real-time END monitoring.
2. Enhanced short-window temporal modeling: we demonstrate that the Transformer-based Self-Attention module effectively captures abrupt and short-lived physiological fluctuations, offering advantages over conventional baselines in short-window temporal modeling.
3. Cost-sensitive learning with dynamic thresholding: by integrating asymmetric cost-sensitive optimization and F1-score-driven threshold tuning, the proposed method effectively addresses severe class imbalance and improves the balance between missed detections and false alarms.
4. Clinically meaningful interpretability: we introduce an IG-based attribution analysis that visually highlights clinically relevant local signal patterns, improving the transparency of model predictions in neurocritical care settings.

## 2 Related work

Early identification of patient deterioration has long been a central topic in intensive care research. To better position the proposed DSF-Net within the existing literature, we review prior work across three key perspectives: single-modality physiological waveform analysis, multimodal temporal modeling, and recent advances in attention-based clinical time-series learning.

Early studies in critical care prediction mainly focused on single-modality physiological waveform analysis. High-frequency waveforms contain rich dynamic information reflecting subtle hemodynamic and electrophysiological changes, and traditional approaches typically relied on handcrafted features combined with conventional machine learning algorithms (6).

With the development of deep learning, 1-dimensional convolutional neural networks (1D-CNNs) have been increasingly used for automated representation learning from raw physiological signals. CNN-based models have shown strong performance in tasks such as arrhythmia detection, cardiac event prediction, and neurological state monitoring. Nevertheless, models based on a single signal source may have limited ability to capture the broader clinical context of neurocritical care patients, where deterioration is often reflected through the interaction of multiple physiological variables rather than a single waveform alone.

To address this limitation, more recent studies have moved toward multimodal physiological fusion, in which continuous waveforms are combined with structured clinical variables such as vital signs or electronic health record data. These approaches provide a more comprehensive representation of patient status and are increasingly used in critical care monitoring. For temporal modeling, recurrent neural networks (RNNs) and long short-term memory (LSTM) networks have been widely adopted to capture sequential dependencies in physiological time series (7). In addition, more complex architectures, such as the Multi-scale deep fusion network, have been proposed for modeling multivariate temporal patterns and anomaly detection in high-dimensional physiological data (8). While these methods are effective in many scenarios, recurrent updates may smooth or dilute short-lived abnormalities, especially when predictive cues appear as abrupt changes within short temporal windows.

More recently, Transformer-based architectures have been introduced into healthcare time-series analysis and have shown promising results in clinical forecasting and representation learning tasks (9). Compared with recurrent models, the Self-Attention mechanism can evaluate all time steps within a sequence in parallel and directly model global temporal dependencies. This property is particularly relevant in our setting, where END precursors may manifest as brief waveform distortions or sudden physiological fluctuations over short windows. In such cases, attention-based modeling may be better suited to highlighting transient abnormalities regardless of their exact position in the sequence. However, despite these methodological advances, applications of attention-based multimodal learning to continuous END prediction in post-stroke neurocritical care remain limited.

Another important gap in the existing literature concerns clinical deployability. Many existing deterioration-prediction models primarily focus on overall classification accuracy, but pay less attention to challenges that are especially important in real-world NCU settings, including severe class imbalance, threshold calibration, false-alarm burden, and interpretability. In practice, deterioration events are rare, and even highly accurate models may become less useful if their decision thresholds are not adapted to local data characteristics or if their outputs cannot be interpreted by clinicians. These limitations motivate the design of our approach.

Against this background, DSF-Net is developed to combine multimodal physiological fusion, short-window temporal modeling, cost-sensitive optimization, and interpretable prediction within a unified framework for continuous END warning.

## 3 Methodology: DSF-Net framework

### 3.1 Overall architecture

**Figure 1:**
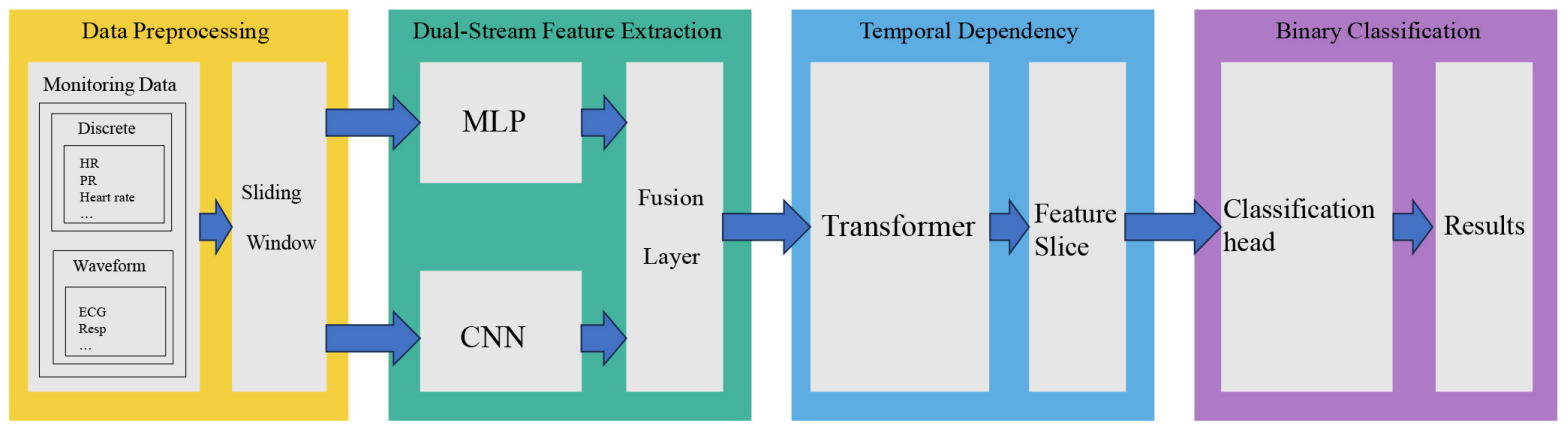
Overall architecture of the DSF-Net framework.

The proposed DSF-Net framework, illustrated in Figure 1, comprises four primary stages: data preprocessing and representation, dual-stream feature extraction, Transformer-based temporal dependency modeling, and binary classification. The pipeline begins by ingesting real-time monitoring data from post-stroke patients in the Neurocritical Care Unit (NCU), organizing it into sliding windows. It then extracts features through a dual-stream architecture—utilizing MLPs for discrete vital signs and CNNs for physiological waveforms—before using them. Finally, a Transformer captures temporal dependencies, leading to a binary classification head that predicts END events.

### 3.2 Data preprocessing and representation

This study utilizes continuous monitoring data acquired from 30 post-stroke patients admitted to the NCU of Tiantan Hospital. The specific data modalities and feature dimensions are detailed in Table 1. For dataset construction, the clinically annotated time of END onset serves as the anchor point. Specifically, data segments falling within a 30-min observation window immediately preceding an END event are labeled as positive instances, whereas segments extracted from stable physiological periods are designated as negative instances.

**Table 1:** Dataset attribute description and feature statistics.

Data category	Feature name/group	Meaning (unit)	Dim.
**Time attribute**	**Time**	**Data acquisition timestamp**	**1**
**Vital signs**	HR, PR	Heart rate, pulse rate (bpm)	2
	SpO_2_, PI	Oxygen saturation (%), Perfusion index (%)	2
	NIBP (S, D, M)	Systolic, Diastolic, Mean pressure (mmHg)	3
	RR	Respiratory rate (RPM)	1
**Cardiac events**	PVCs, VPBs	Ventricular premature beats, premature ventricular contractions (/min)	2
	Pauses, Missed	Heart pauses, Missed beats (/min)	2
	Couplets	Couplet ventricular premature beats (/min)	1
	R on T	R-on-T phenomenon occurrences (/min)	1
**Physiological waveforms**	ECG_[0–255]	Electrocardiogram signal sample sequence	256
	Resp_[0–255]	Respiration waveform sample sequence	256
	Pleth_[0–59]	Finger pulse oximetry volume waveform sample sequence	60
**Total**	**—**	**Overall dimension of all input features**	**587**

Bold text indicates feature category/group labels and the overall total feature dimension.

To precisely capture transient pathological patterns within continuous physiological streams, we employ a high-overlap sliding-window sampling strategy. The sampling window length is set to *L* = 15 s, which covers approximately 15–25 complete cardiac cycles from an electrophysiological perspective, preserving the morphology evolution of the P-QRS-T complex and short-term frequency domain characteristics of heart rate variability (HRV). A stride of *S* = 1 s is applied to the sliding window, yielding an overlap ratio of approximately 93%. This dense sampling strategy is implemented for two primary purposes:

- Enhanced sensitivity: it allows the model to detect subtle physiological precursors to acute pathologies, such as clusters of ventricular premature beats or R-on-T phenomena.
- Temporal granularity: by minimizing the risk of missing fleeting abnormal signals in the time dimension, it provides fine-grained input sequences that enable the subsequent Transformer module to effectively capture long-range temporal dependencies.

Given the substantial data volume generated by the high overlap approach, memory mapping techniques are utilized to optimize I/O efficiency, ensuring that sampling resolution is maintained without compromising model training performance.

### 3.3 Dual-stream feature extraction

#### 3.3.1 Waveform CNN processing module

For high-frequency physiological waveforms (ECG, Resp, and Pleth, as detailed in Table 2), a 1D-CNN-based encoder is designed to extract latent morphological features. First, the Pleth signal (originally sampled at a lower rate) is upsampled via linear interpolation to match the temporal resolution of the other signals (256 points). These three synchronized waveforms are then concatenated to form an input tensor of shape (3, 256). The encoder consists of two sequential convolutional blocks, each block utilizing a one-dimensional convolution kernel of size 7 and stride 2 followed by Batch Normalization and ReLU activation. Additionally, a Dropout layer (*p* = 0.3) is incorporated to enhance model generalization and mitigate overfitting. Following the two levels of convolutional downsampling, the feature maps are flattened, mapping the original time-domain signals into a 2,048-dimensional high-level feature space. This vector serves as a compressed representation of the single-frame physiological state, which is then fed into the subsequent modality fusion module.

**Table 2:** Model performance comparison.

Model	AUC	Recall	F1-score	Optimal threshold
**DSF-Net (Ours)**	**0.9996**	**0.9960**	**0.9841**	0.9792
LSTM	0.9312	0.7410	0.7579	0.9804
ResNet 1D	0.9381	0.3233	0.1453	0.8874
MS-CRED	0.5160	0.8267	0.0143	N/A

Bold values indicate the proposed model and the best performance values among the compared models.

#### 3.3.2 Numerical MLP processing module

Complementing the raw physiological waveforms, clinical monitoring also includes several key discrete vital sign parameters, such as heart rate (HR), pulse rate (PR), oxygen saturation (SpO_2_), blood pressure (NIBP), and respiratory rate (RR), which reflect the patient's autonomic nervous regulation status and circulatory stability. These numeric features, though low-dimensional, have clear clinical interpretability, and their dynamic changes often indicate underlying pathological compensation mechanisms.

To fully exploit the non-linear interactions among these variables, a lightweight multilayer perceptron (MLP) is designed as the numerical feature encoder. This module takes the 14-dimensional vector from each time frame (see Table 2) as input and models physiological correlations through two fully connected layers, each followed by Batch Normalization and ReLU activation. Significantly, the network projects the original observations into a 32-dimensional latent embedding. This step balances the feature representation, preparing it for integration with the high-dimensional waveform features. Finally, the MLP-encoded numerical vector is concatenated with the 2,048-dimensional CNN output, resulting in a 2,080-dimensional joint representation vector. This fusion strategy effectively preserves the fine-grained morphological details of the waveforms while incorporating clinically significant quantitative indicators.

### 3.4 Temporal dependency modeling based on transformer

For early END events that may occur after endovascular treatment for acute ischemic stroke, traditional static risk scoring systems are inadequate for continuous monitoring-based dynamic alert tasks. END often arises from the cumulative effect of multiple latent pathological processes (such as reperfusion injury, progressive cerebral edema, or secondary microcirculatory disorders) (10), with precursor signals frequently appearing in a subtle and intermittent manner within long sequences of physiological data. Therefore, this study constructs a sliding window-based temporal modeling mechanism to identify predictive temporal evolution patterns from high-frequency multi-modal monitoring data.

First, the model acquires the morphological and physiological state representations at the current time point through the CNN and MLP branches, respectively, at each 1-s sampling point. Subsequently, the frame-level features from consecutive *T* = 15 time steps are concatenated and linearly projected into a 128-dimensional shared embedding space. To preserve the temporal order in physiological signals, learnable position encodings are injected into the sequence.

The core modeling layer employs a Transformer Encoder architecture, utilizing its multi-head self-attention mechanism to aggregate context within the 15-frame sliding window, thereby capturing long-term dependencies related to disease precursors. Considering the causality and timeliness of real-time alerts, this study adopts a “last-step pooling” strategy, extracting only the hidden state of the last time step in the Transformer output sequence as the global representation of the current window. This representation encapsulates historical trend information, which is then mapped to a binary prediction of whether an END might occur through a fully connected classification head.

This hierarchical modeling architecture effectively addresses the limitations of traditional methods that rely solely on instantaneous thresholds while ignoring dynamic trends, significantly enhancing the model's ability to recognize rare pathological events.

## 4 Benchmark models

To objectively assess the efficacy of the proposed DSF-Net architecture in multimodal temporal modeling, three representative benchmark models were selected for comparison:

1. **ResNet-1D (convolutional benchmark)**: aimed at evaluating the ability of one-dimensional convolutional networks based on deep residuals to capture morphological features of physiological waveforms, exploring performance bottlenecks of local feature extractors in the absence of global temporal modeling.
2. **LSTM (recurrent benchmark)**: a classical recurrent neural network, used to gauge the discriminative accuracy of traditional temporal models when handling long-range physiological dependencies.
3. **MS-CRED** **(****11****)** **(multi-scale attention-based benchmark)**: a sophisticated encoder-decoder framework that utilizes multi-scale signature matrices to capture inter-sensor correlations and temporal dynamics. This model serves as a representative of state-of-the-art anomaly detection methods that combine convolutional and recurrent structures for complex multivariate time series.

To ensure fairness in the comparison results, all benchmark models were trained under identical preprocessing procedures, hardware environments, and cost-sensitive loss function configurations. This rigor ensures that any observed differences in performance can be attributed solely to disparities in architectural design.

## 5 Case study

### 5.1 Experimental setup and hyperparameter configuration

All experiments in this paper were conducted on an NVIDIA GeForce RTX 3080 GPU platform, augmented with parallel computing acceleration. The raw dataset was partitioned into training, validation, and independent test sets at a ratio of 8:1:1 with strict patient-level isolation. To address the issue of class imbalance, a cost-sensitive learning strategy was employed. Specifically, the weight coefficient ω_*pos*_ of the loss function (BCEWithLogitsLoss) was set to 160. The model was optimized using the Adam optimizer with a fixed learning rate of 1 × 10^−4^ and a batch size of 4,096. Regarding the model architecture, the sliding window length was fixed at *T* = 15. At each time step, the 2080-dimensional fused feature vector (derived from the waveform and numerical branches) was linearly projected into a 128-dimensional latent space before serving as input to the Transformer encoder. The training process spanned a maximum of 50 epochs, incorporating an early stopping mechanism that automatically saves the optimal model parameters based on the minimum validation loss.

### 5.2 Evaluation criteria

To assess the generalization performance of the model on the patient-level independent test set, this study adopted a suite of metrics suitable for asymmetrically distributed data. In addition to AUC, emphasis was placed on Recall and F1-Score to characterize the model's capability in detecting rare positive events. Relevant metrics are defined as follows:

1. **Recall (recall rate):**
$$\begin{array}{l} \text{Recall}=\frac{TP}{TP+FN} \end{array}$$ (1)
  in Equation 1 where *TP* denotes true positives (True Positives, TP), i.e., the number of samples correctly predicted as positive by the model; *FN* represents false negatives (False Negatives, FN), i.e., the number of positive samples incorrectly predicted as negative by the model. In clinical alert scenarios, Recall is also known as Sensitivity, serving as a core indicator for assessing the risk of missed diagnoses.
2. **F1-Score:**
$$\begin{array}{l} F_{1}=2\cdot \frac{\text{Precision}\cdot \text{Recall}}{\text{Precision}+\text{Recall}}=\frac{2\cdot TP}{2\cdot TP+FP+FN} \end{array}$$ (2)
  in Equation 2 where Precision represents precision, i.e., the proportion of actual positive samples among those predicted as positive by the model.
3. **Area Under Curve (AUC):**
$$\begin{array}{l} \text{AUC}=\frac{\sum_{i\in \text{pos}}{\text{rank}}_{i}-\frac{M\left(M+1\right)}{2}}{M\times N} \end{array}$$ (3)
  in Equation 3 where:
  - *M* denotes the total number of positive samples (Positive samples);
  - *N* represents the total number of negative samples (Negative samples);
  - rank_*i*_ indicates the rank of the *i*th positive sample when sorted in ascending order by predicted probability;
  - $\underset{i\in \text{pos}}{\sum }$ signifies the summation of ranks for all positive samples.
4. **Weighted binary cross-entropy loss:** to mitigate gradient dilution caused by the extreme class imbalance of 1:160, a cost-sensitive loss function was employed during training in this study.
$$\begin{array}{l} L=-\frac{1}{N}\overset{N}{\underset{i=1}{\sum }}\left[\omega \cdot y_{i}\log\left(p_{i}\right)+\left(1-y_{i}\right)\log\left(1-p_{i}\right)\right] \end{array}$$ (4)
  in Equation 4 where:
  - *y*_*i*_∈{0, 1} is the true label of sample *i*;
  - *p*_*i*_∈[0, 1] is the predicted probability output by the model;
  - ω is the weighting coefficient for positive samples (set to 160 in this study), explicitly increasing the penalty cost for missed diagnoses (FN) by the model.

Considering that the 1:160 class imbalance would cause the model's output probability distribution to skew toward the majority class, this study abandoned the conventional fixed threshold of 0.5 and instead adopted a decision threshold optimization strategy. The criterion for selecting the optimal threshold τ^*^ is given by Equation 5:


$$\begin{array}{l} \tau^{*}=\arg\underset{\tau \in \left(0,1\right)}{\max}F_{1}\left(\tau \right) \end{array}$$ (5)

This method scans the probability space on the validation set, identifying the operating point that maximizes F1-Score, thus achieving a Pareto-optimal balance between the risk of missed diagnoses and the frequency of false alarms.

### 5.3 Comparative experiments

ResNet-1D, LSTM, and MS-CRED were introduced for comparative experiments to evaluate the performance metrics of DSF-Net. Results are presented in Table 2. Note that for MS-CRED, the optimal threshold is marked as N/A as it operates on a fixed reconstruction error strategy for anomaly detection rather than a probabilistic classification threshold, making direct threshold-based optimization inapplicable in this comparative setting.

The experimental results demonstrate that, under the severe class imbalance ratio of 1:160, DSF-Net exhibits exceptional discriminative performance. Compared to traditional sequential models (LSTM), residual networks (ResNet-1D), and the multi-scale correlation-based model (MS-CRED), DSF-Net maintains a remarkable AUC of 0.9996 and achieves an F1 score of 0.9841.

In particular, the MS-CRED model, despite its sophisticated multi-scale signature matrix design, failed to achieve satisfactory results in this specific clinical context, yielding an AUC of only 0.5160 and an F1 score of 0.0143. While MS-CRED showed a relatively high recall (0.8267), its extremely low precision (0.0072) suggests that it struggled to distinguish true physiological precursors from noise under extreme class disparity, leading to an overwhelming number of false positives. Similarly, the ResNet-1D baseline achieved an F1 score of only 0.1453, further highlighting the limitations of traditional architectures.

These findings indicate that DSF-Net effectively captures the subtle physiological precursors associated with rare positive samples. In contrast, baseline models exhibit severe prediction degradation or excessive sensitivity when confronted with such extreme class imbalance. Ultimately, these results validate the robustness and effectiveness of the proposed architecture in handling complex, imbalanced clinical data.

### 5.4 Interpretability

**Figure 2:**
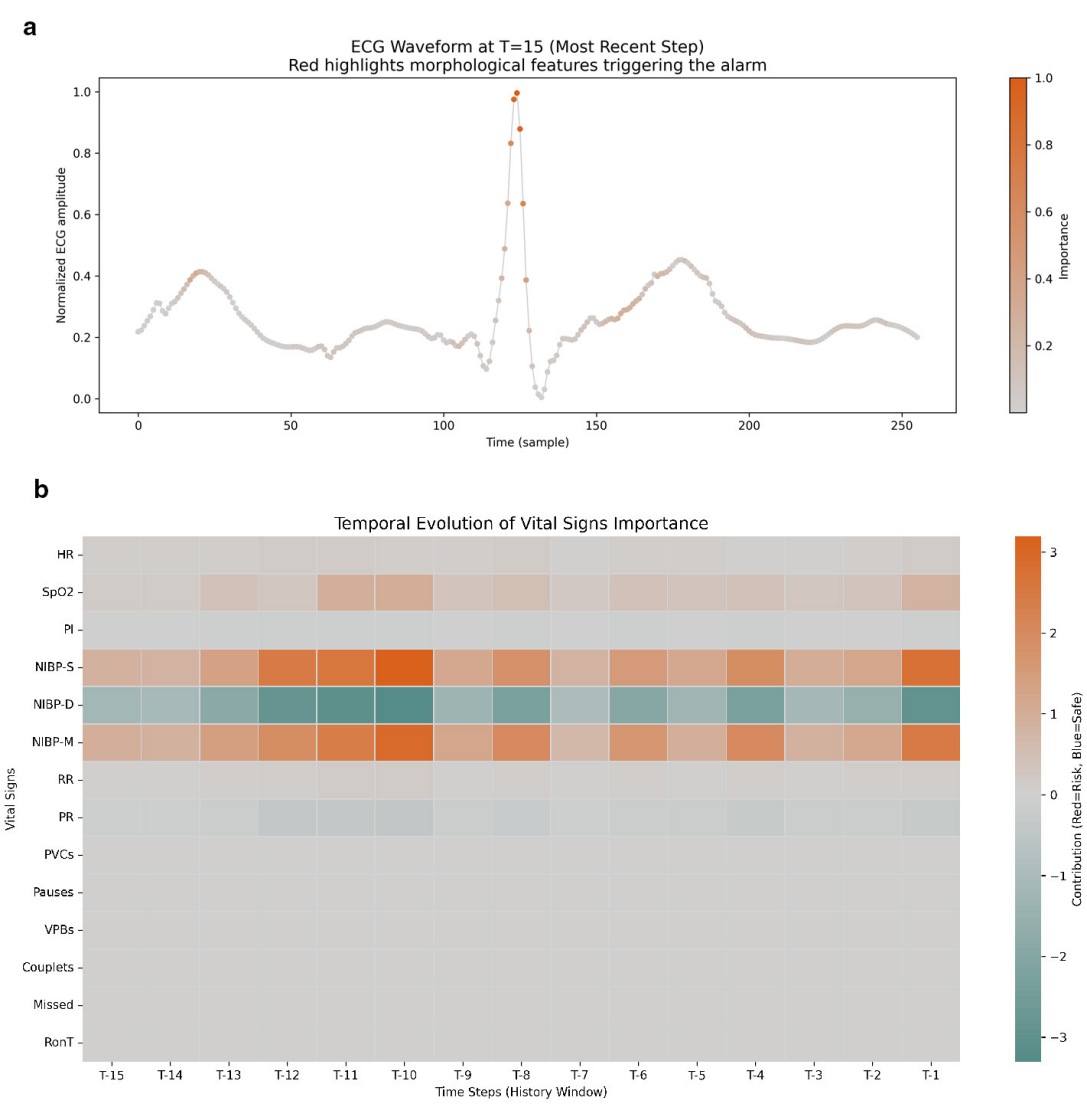
Model interpretability scheme using Integrated Gradients (IG). **(a)** Projection of attribution weights onto physiological waveforms to highlight pathological substructures. **(b)** A 15-s temporal heatmap where orange shades represent risk-driving effects and green shades represent stabilizing effects of vital signs.

To enhance decision transparency in clinical settings, this study employs the Integrated Gradients (IG) algorithm (12) for feature attribution analysis. Unlike perturbation-based local explanation methods, IG satisfies the axioms of Sensitivity and Implementation Invariance, making it more suitable for robust feature attribution in high-dimensional temporal feature spaces of nonlinear deep networks. Given the heterogeneity of the model fusing waveform and numerical indicators, we devise an intuitive explanation scheme (see Figure 2).

The model precisely locates pathological features by projecting attribution weights back onto the original physiological waveforms. As shown in Figure 2a, IG accurately highlights key substructures within the waveforms (such as QRS complex morphology variations or potential shifts in specific segments). This waveform feature significance visualization allows clinical experts to verify whether the signal abnormalities captured by the model align with established electrophysiological and pathophysiology principles, thereby validating the reliability of the alerts.

For discrete vital signs, we use a temporal contribution heatmap to assess the dynamic influence of each indicator within a 15-s observation window. As depicted in Figure 2b, orange shades represent a positive driving effect (increasing risk probability) of the indicator, while green shades indicate a negative suppressive effect (maintaining stability). This heatmap clearly distinguishes whether the alert is triggered by an acute physiological fluctuation (e.g., a sudden drop in SpO_2_) or by cumulative deterioration of multiple indicators, providing direct evidence for prioritizing clinical interventions.

### 5.5 Score distribution analysis

**Figure 3:**
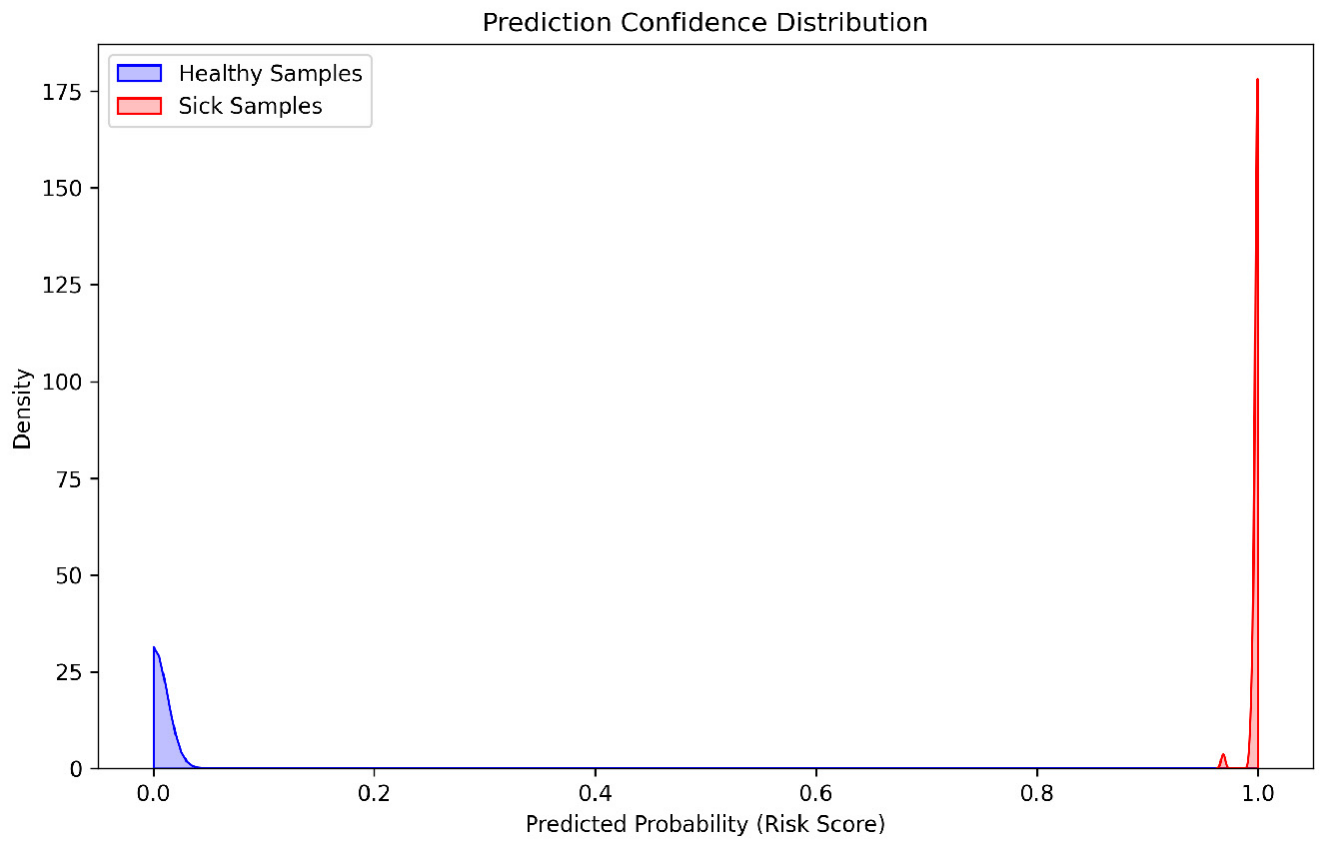
Probability density distribution.

To visually assess the model's robustness in discriminating target events, we plot the probability density distributions of negative samples and high-risk samples (see Figure 3). The results show that the two types of samples exhibit distinct bimodal distribution characteristics in probability space: Healthy samples (blue) are densely concentrated in the near-zero probability range, while positive samples (red) tightly cluster in the high probability interval (≈1). This excellent inter-class separation indicates that, even when confronted with the extreme distribution challenge of 1:160, the model can effectively establish a decisive decision boundary. The near-absence of overlap between the two distributions not only validates the model's precision in extracting physiological features but also corroborates the nearly perfect AUC score achieved on the test set, reflecting the system's low false alarm risk and high discrimination credibility in real-time monitoring.

### 5.6 Ablation study

To assess the contribution of each component in the proposed framework to the prediction performance, a series of ablation experiments were conducted on the original model. Three ablation variant models were designed for comparative experiments. Under the premise of maintaining the same hyperparameter settings, each variant selectively removed different parts of the model. The detailed configurations are presented in Table 3. Table 4 presents the quantitative results of the ablation experiments.

**Table 3:** Ablation experiment configurations.

Model	Architecture Modifications
**Full model**	**Complete dual-stream temporal model (baseline)**
w/o Waveform branch	Removes the waveform CNN processing module, relying solely on numerical features
w/o Temporal fusion	Removes the transformer encoder, utilizing only the final frame's features for classification
w/o Positional encoding	Removes temporal position encoding, retaining only the attention pooling mechanism

Bold text indicates the full baseline model configuration; the remaining rows correspond to ablated variants.

**Table 4:** Model performance comparison.

Model	Val loss	AUC	Recall	F1	Threshold
**Full model (baseline)**	**0.0025**	0.9996	**0.9960**	**0.9841**	0.9792
w/o Waveform Branch	0.0106	**0.9999**	0.9666	0.9530	0.9949
w/o Temporal Fusion	0.0429	0.9995	0.9035	0.8850	0.9861
w/o Positional Encoding	0.0363	0.9993	0.9158	0.8528	0.9931

Bold values indicate the best result in each metric column, where lower is better for validation loss and higher is better for AUC, recall, and F1.

The removal of the waveform feature extraction branch (w/o Waveform Branch) led to a decrease in F1 score by approximately 3.1%. This indicates that while discrete indicators provide basic pathological information, the subtle morphological features embedded in high-frequency waveforms play an indispensable role in precisely capturing critical illness risk. The combination of waveforms and numerical indices constructs a more comprehensive feature space.

Eliminating the Transformer temporal fusion module (w/o Temporal Fusion) resulted in an F1 score of 0.8850. This finding underscores the insufficiency of single-frame predictions in capturing the dynamic evolution of critical illnesses. Compared to the full model, this variant exhibits the most severe performance degradation, verifying the dominant role of long-range temporal dependencies in early warning tasks.

Upon removing position encoding (w/o Positional Encoding), the model's F1 score decreased to 0.8528. This reflects the crucial importance of event sequence in medical data for the model's discriminative ability. The lack of position awareness causes the attention mechanism to degenerate into a “bag-of-feature” approach, losing its capacity to model the logical progression of physiological signals.

### 5.7 Robustness analysis and overfitting verification

To evaluate the generalization capability of our proposed model and ensure that the performance of the model was not a result of overfitting or data leakage, we implemented a comprehensive evaluation protocol. This evaluation protocol utilized a five-fold cross-validation (CV) approach (13) conducted on 90% of all the samples, followed by a final assessment on a completely independent holdout test set consisting of the remaining 10% of all the samples. The sets were partitioned strictly based on unique Patient IDs, ensuring that data from any individual patient was confined exclusively to a single fold or the holdout test set. This patient-level isolation strictly precludes inter-set data leakage and ensures that the model's predictive performance is evaluated on entirely unfamiliar subjects.

As illustrated in Table 5, the model exhibited exceptional stability across all five folds. The best validation AUC for each fold ranged from 0.9990 to 0.9995. Throughout the 20 epochs of training per fold, the AUC scores demonstrated a consistent upward trajectory, starting from approximately 0.90 in the first epoch and converging toward 0.999 by the final epoch. The negligible standard deviation in performance across different data partitions indicates that the model is highly robust to variations in data distribution.

**Table 5:** Detailed performance metrics for five-fold cross-validation and holdout testing.

Fold ID	Best Val. AUC	Val. recall	Holdout test AUC
Fold 1	0.9995	0.9980	0.9996
Fold 2	0.9992	0.9989	0.9992
Fold 3	0.9990	0.9980	0.9990
Fold 4	0.9992	0.9996	0.9993
Fold 5	0.9994	0.9993	0.9994
**Average**	**0.9993**	**0.9988**	**0.9993**

Bold values indicate the average performance across the five cross-validation folds.

The “Generalization Gap,” also known as the difference between the validation performance during CV and the performance on the unseen holdout test set, is the primary indicator of overfitting. In our experiments, the average best validation AUC across the five folds was 0.9993. When these fold-specific models were applied to the holdout test set, they achieved an identical average AUC of 0.9993.

The achievement of near-zero mean generalization gap (Δ*AUC* < 0.0001) on the dataset provides strong evidence that the model has successfully learned the underlying physiological representations of END rather than memorizing noise or specific samples. Also, the temporal separation and patient-level splitting protocols effectively prevented any form of data leakage. Last, the high sensitivity maintained alongside high AUC scores demonstrates the model's effectiveness in handling the extreme class imbalance present in clinical monitoring data.

## 6 Discussion

The strong predictive performance and the 30-min lead time achieved by our model may have important clinical implications for neurocritical care. In patients at risk of END, even a short advance warning may provide clinicians with a valuable opportunity to reassess the patient, initiate additional monitoring, and consider early intervention before irreversible secondary injury occurs. In this context, the ability of the model to maintain high sensitivity while preserving a low false-alarm burden is especially relevant, since excessive false alerts can reduce clinician trust and contribute to alarm fatigue in intensive care environments.

An important aspect of clinical translation is the maintenance of the decision threshold (τ^*^) over time. In real-world NCU settings, a fixed threshold may become suboptimal as a result of concept drift, including shifts in patient characteristics, monitoring practices, case mix, or institutional treatment protocols. For this reason, a practical deployment strategy may benefit from periodic threshold recalibration based on recent local data. For example, the operating threshold could be re-estimated at regular intervals using recent retrospective cases while monitoring false-alarm and missed-detection rates. Although such a recalibration mechanism was not prospectively evaluated in the present study, we believe it represents an important step toward maintaining a clinically appropriate balance between sensitivity and false alarms across changing care environments.

The present results also provide preliminary evidence regarding model generalization. Despite the relatively specialized cohort, the close agreement between cross-validation performance and the independent holdout test suggests that the model is learning stable and clinically meaningful physiological patterns associated with deterioration. In addition, all experiments were conducted using strict patient-level partitioning, which reduces the risk of overlap-induced leakage and supports a more rigorous assessment of generalization. The stronger performance of DSF-Net relative to baseline models such as LSTM and MS-CRED further suggests that combining high-frequency waveform information with structured vital-sign features is beneficial for END prediction. Consistent with the case analysis, these findings support the interpretation that the model captures physiologically relevant waveform and vital-sign changes preceding deterioration, rather than simply memorizing patient-specific signal patterns.

Several limitations should also be acknowledged. First, this study was conducted on a single-center cohort, and the number of unique patients remains limited despite the dense high-frequency sampling. Therefore, caution is warranted when interpreting the broader generalizability of the model. Second, although we discuss threshold recalibration as a practical strategy for clinical deployment, this mechanism has not yet been prospectively validated in a live NCU environment. Future work will focus on multi-center external validation, inclusion of more diverse clinical variables, and prospective assessment of threshold maintenance strategies under real-world workflow conditions. These efforts will be important for further evaluating the robustness, portability, and clinical utility of DSF-Net.

## 7 Conclusion

This study proposes an interpretable streaming early warning model for acute critical events based on the synergistic fusion of electrocardiogram waveforms and vital signs, abbreviated as DSF-Net. Innovatively, the model introduces a dual-stream architecture to concurrently analyze continuous waveforms and discrete physiological metrics, effectively capturing complementary feature representations to enhance prediction accuracy. To address the challenge of extreme data imbalance, a cost-sensitive learning mechanism is integrated into the training process. This strategy significantly mitigates the bias toward the majority class, thereby reducing misdiagnosis rates and outperforming traditional baselines in identifying rare pathological events. Furthermore, the incorporation of an IG-based attribution algorithm provides crucial model transparency. By visually pinpointing the specific temporal and morphological locations of abnormal physiological signals, it provides diagnostic interpretability and furnishes more reliable evidence for clinical decision-making.

## Data Availability

The data analyzed in this study is subject to the following licenses/restrictions. Requests to access the datasets should be directed to the corresponding author, vip2ljj@163.com.
